# Chronic inhibition of tumor cell-derived VEGF enhances the malignant phenotype of colorectal cancer cells

**DOI:** 10.1186/1471-2407-13-229

**Published:** 2013-05-07

**Authors:** Naoko Yamagishi, Shigetada Teshima-Kondo, Kiyoshi Masuda, Kensei Nishida, Yuki Kuwano, Duyen T Dang, Long H Dang, Takeshi Nikawa, Kazuhito Rokutan

**Affiliations:** 1 Department of Stress Science, Institute of Health Biosciences, University of Tokushima Graduate School, Tokushima 770-8503, Japan; 2 Department of Physiological Nutrition, Institute of Health Biosciences, University of Tokushima Graduate School, 3-18-15 Kuramoto-cho, Tokushima, 770-8503, Japan; 3 Division of Gastroenterology, Department of Internal Medicine, University of Michigan, Ann Arbor, MI, 48109, USA; 4 Division of Hematology/Oncology, Department of Internal Medicine, University of Florida Shands Cancer Center, University of Florida, 1600 SW Archer Road, Gainesville, FL, 32610, USA

## Abstract

**Background:**

Vascular endothelial growth factor-a (VEGF)-targeted therapies have become an important treatment for a number of human malignancies. The VEGF inhibitors are actually effective in several types of cancers, however, the benefits are transiently, and the vast majority of patients who initially respond to the therapies will develop resistance. One of possible mechanisms for the acquired resistance may be the direct effect(s) of VEGF inhibitors on tumor cells expressing VEGF receptors (VEGFR). Thus, we investigated here the direct effect of chronic VEGF inhibition on phenotype changes in human colorectal cancer (CRC) cells.

**Methods:**

To chronically inhibit cancer cell-derived VEGF, human CRC cell lines (HCT116 and RKO) were chronically exposed (2 months) to an anti-VEGF monoclonal antibody (mAb) or were disrupted the *Vegf* gene (VEGF-KO). Effects of VEGF family members were blocked by treatment with a VEGF receptor tyrosine kinase inhibitor (VEGFR-TKI). Hypoxia-induced apoptosis under VEGF inhibited conditions was measured by TUNEL assay. Spheroid formation ability was assessed using a 3-D spheroid cell culture system.

**Results:**

Chronic inhibition of secreted/extracellular VEGF by an anti-VEGF mAb redundantly increased VEGF family member (PlGF, VEGFR1 and VEGFR2), induced a resistance to hypoxia-induced apoptosis, and increased spheroid formation ability. This apoptotic resistance was partially abrogated by a VEGFR-TKI, which blocked the compensate pathway consisted of VEGF family members, or by knockdown of *Vegf* mRNA, which inhibited intracellular function(s) of all *Vegf* gene products. Interestingly, chronic and complete depletion of all *Vegf* gene products by *Vegf* gene knockout further augmented these phenotypes in the compensate pathway-independent manner. These accelerated phenotypes were significantly suppressed by knockdown of hypoxia-inducible factor-1α that was up-regulated in the VEGF-KO cell lines.

**Conclusions:**

Our findings suggest that chronic inhibition of tumor cell-derived VEGF accelerates tumor cell malignant phenotypes.

## Background

Angiogenesis is a key event in the process of tumor growth and metastasis. The well-established role of vascular endothelial growth factor-a (VEGF) in tumor angiogenesis has led to the development of therapeutic strategies that selectively target the VEGF pathway. Therefore, anti-VEGF therapies were initially proposed for inhibiting solid tumors. It was thought that such therapies would be less susceptible to resistance given the target was genetically stable tumor endothelial cells as opposed to genetically unstable cancer cells. Drugs that target VEGF or the VEGF receptors (VEGFR) have been shown to prolong survival in patients with several cancer types, including metastatic colorectal cancer (CRC) [1]. However, now after several years of anti-VEGF therapies being used in patients with solid tumors, it has become clear that most of patients, regardless of their tumor type, will ultimately exhibit resistance to VEGF-targeted therapy. Mechanisms of the resistance include up-regulation of alternative proangiogenic factors, protection of the tumor vasculature either by recruiting proangiogenic proinflammatory cells or by increasing protective pericyte coverage, and accentuated invasiveness of tumor cells into local tissue to co-opt normal vasculature [2-6]. In addition to these proposed mechanisms, oncologists have begun to focus on the mechanisms of direct action of anti-VEGF agents on cancer cells and tumor adaptation to VEGF inhibition [2,3].

In fact, VEGFR is expressed not only in endothelial cells but also in several cancer cell lines, including CRC, bladder, breast, and pancreatic cancer cells [7-10]. In addition, an immunohistochemical screen of non-endothelial cancer specimens revealed detectable levels of VEGFR in CRC, bladder, breast, and lung cancers [10]. These observations suggested a possible autocrine/paracrine VEGF signaling pathway within cancer cells. In fact, it has become clear that VEGF acts as an autocrine growth and survival factor for cancer cells that express VEGFR [8-10]. Some of the effects observed with anti-VEGF therapies may therefore result from “direct” effects on tumor cells, i.e., actions that are independent of the antiangiogenic effects of VEGF inhibitors. Several reports have now shown that the loss of VEGF signaling in cancer cells, induced by either VEGF pathway targeting agents or *Vegf* gene disruption, facilitates migration, invasion and metastasis of tumor cells *in vitro* and *in vivo*[11-13]. Particularly in the *in vivo* situation, anti-VEGF therapies may synergistically promote tumor cell malignancy not only by direct action on tumor cells but also through the indirect effect of inducing tumor hypoxia [14].

However, the direct effects of anti-VEGF therapy on tumor cells under hypoxic conditions are not yet fully understood. In this study, we evaluated the direct effects of not only chronic blockade of secreted/extracellular VEGF but also chronic loss of all of *Vegf* gene products on tumor cell phenotypes under hypoxic conditions *in vitro*. We found that chronic exposure of CRC cells to an anti-VEGF monoclonal antibody (anti-VEGF mAb; the mAb-long cells) *in vitro* resulted in a resistance to hypoxia-induced apoptosis and an increased spheroid formation ability. These phenotypic alterations were partially suppressed by treatment with a VEGFR-TKI or by knockdown of *Vegf* mRNA that could inhibit intracellular *Vegf* gene products, including the 5′UTR of *Vegf* mRNA [15] and/or intracrine VEGF [16]. Furthermore, chronic depletion of all *Vegf* gene products by *Vegf* gene knockout (VEGF-KO) augmented these phenotypes. Hypoxia-inducible factor-1α (HIF-1α) contributed in the phenotype of the VEGF-KO cells as well as the mAb-long cells. These results provide a new insight into the adaptation of CRC cells to the loss of VEGF.

## Methods

### Cell culture, transfection and treatment

Human colon cancer cell lines (HCT116 and RKO) were maintained in McCoy’ s 5A medium with 10% fetal bovine serum and antibiotics. Transfection of cells with plasmid was performed using the JetPEI transfection regent (Polyplus-transfection, Illkirch, France), according to the manufacture’s instructions. Cells were treated with anti-human VEGF mAb (5 μg/ml, R & D systems) or VEGFR tyrosine kinase inhibitor III that inhibits VEGFR-1, -2 and -3 (0.36 μM, KRN633, Calbiochem).

### Development of the mAb-long cell lines

HCT116 and RKO cells were exposed to anti-human VEGF mAb (5 *μ*g/ml) for 2 months *in vitro* to develop the mAb-long cell lines. HCT116 and RKO cells were also exposed to non-immune mouse IgG (5 *μ*g/ml) in parallel to generate the control IgG-long cell lines.

### Hypoxic treatment and HIF-1α-dependent transcriptional activity

For hypoxic culture conditions, cells were incubated at low confluence and 37°C in BBL GasPak 100 anaerobic system in which O_2_ was ~0.1% (BD Biosciences). Hypoxic treatment was functionally confirmed by transactivation of HIF-1α using a HIF-1α-dependent reporter construct combined with internal control reporter construct (Cignal HIF reporter assay kit, SA Biosciences).

### Quantitative RT-PCR (qRT-PCR)

The levels of transcripts for VEGF ligands (*Vegf-a*, *Vegf-b*, and *Plgf*), VEGF receptors (*Vegfr1* and *Vegfr2*),, *Hif-1α*, and β-actin were measured by real time (RT)-PCR using the following specific primer sets: *Vegf-a*, 5′- GAGCCTTGCCTTGCTGCTCTAC -3′ (forward) and 5′- CACCAGGGTCTCGATTGGATG -3′ (reverse); *Vegf-b*, 5′- CTGGCCACCAGAGGAAAGT -3′ (forward) and 5′- CATGAGCTCCACAGTCAAGG -3′ (reverse); *Plgf*, 5′- GGCTGTTCCCTTGCTTCC -3′ (forward) and 5′- CAGACAAGGCCCACTGCT -3′ (reverse); *Vegfr1*, 5′- AGAACCCCGATTATGTGAGAAA -3′ (forward) and 5′- GATAGATTCGGGAGCCATCC -3′ (reverse); *Vegfr2*, 5′- GAACATTTGGGAAATCTCTTGC -3′ (forward) and 5′- CGGAAGAACAATGTAGTCTTTGC -3′ (reverse); *Hif-1α*, 5′- CAGCTATTTGCGTGTGAGGA -3′ (forward) and 5′- TTCATCTGTGCTTTCATGTCATC -3′ (reverse); *HuR*, 5′- CCAGGCGCAGAGATTCAG -3′ (forward) and 5′- GGTTGTAGATGAAAATGCACCAG -3′ (reverse); β-actin, 5′- CCAACCGCGAGAAGATGA -3′ (forward) and 5′- CCAGAGGCGTACAGGGATAG -3′ (reverse). Amplification and quantification of the PCR products were performed using the Applied Biosystems 7500 System (Applied Biosystems). Standards were run in the same plate and the relative standard curve method was used to calculate the relative mRNA expression. RNA amounts were normalized against the β-actin mRNA level.

### Measurement of VEGF promoter activity

Cells were cotransfected with the reporter plasmid containing the promoter of VEGF (-2362 to +90 nt sequence of human *Vegf* gene) and a pRL-TK plasmid (as a monitor for transfection efficiency). Reporter mRNA levels were measured using qRT-PCR and normalized to the levels of Renilla luciferase mRNA from a pRL-TK plasmid.

### siRNA and transfection

Stealth RNAi negative control siRNA (medium GC content, Invitrogen) was used as a control siRNA, which has no homology to human gene products. The siRNA targeting *Vegf* mRNA duplex targets 5′- GAUCUCAUCAGGGUACUCC-3′ (B-Bridge Int.). The siRNA targeting *Hif-1α* mRNA targets 5′- CCUCAGUGUGGGUAUAAGA -3′ (Ambion). The siRNAs targeting the *HuR* mRNA targets 5′-AAGAGGCAATTACCAGTTTCA-3′ (Ambion). Cells were transfected with siRNA using Lipofectamine RNAiMax reagent (Invitrogen), according to the manufacturer’s instructions. Transfection efficiency of siRNA was approximately 60-70% that was determined using BLOCK-iT Alexa Fluor Red Fluorescent Oligo (Invitrogen).

### Assessment of apoptosis

Apoptotic cells were assessed by a DeadEnd TUNEL-staining kit (Promega), as previously described [15].

### mRNA stability analysis

mRNA stability was determined by actinomycin D experiments. Briefly, control and HuR-silenced cells were treated with actinomycin D (2 μg/ml) to block further transcription. At 8 h after actinomycin D treatment under hypoxic conditions, the cells were harvested and mRNA was quantified by RT-qPCR. The mRNA decay was recorded as the percentage of mRNA remaining over time compared with the amount before the addition of actinomycin D.

### Spheroid formation assay

Two-hundred microliter cell suspension with 5 × 10^3^ cells were seeded into each well of a 96-well NanoCulture plate (SCIVAX, Inc., Japan). The plates were incubated at 37°C in 5% CO_2_. On day 3, loose spheroids had formed and 100 μl/well of medium was replaced with fresh medium. After another 3 days of culture, larger and tighter 6 day spheroids had formed.

### Statistical analysis

Results are expressed as means ± S.D. Statistical analyses of data were done using ANOVA and the Scheffé’s test. P values < 0.05 was considered significant.

## Results

### Effect of chronic VEGF inhibition on the expression of VEGF family members

**Figure 1:**
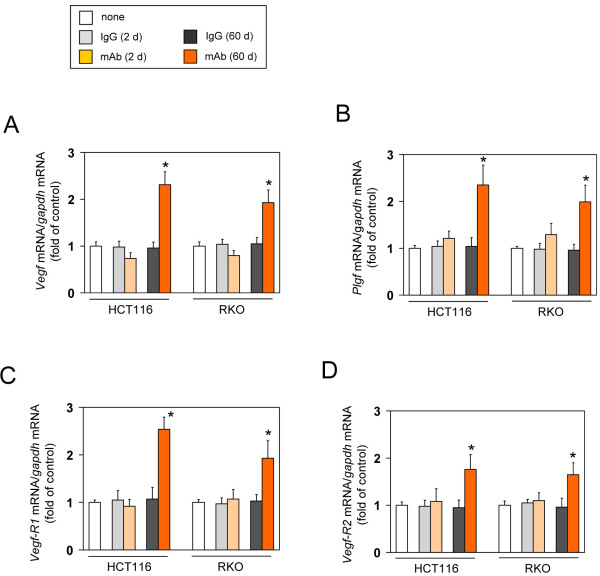
**Effect of inhibition of cancer cell-derived secreted VEGF on VEGF family profile.** Cells were treated without (none) or with an unimmunized IgG or an anti-VEGF mAb for 2 or 60 days. Expression levels of *Vegf *(**A**), *Plgf *(**B**), *Vegfr1 *(**C**), and *Vegfr2 *(**D**) were measured by quantitative RT-PCR (n=4, means ± S.D.). *P < 0.01.

We first examined whether acute or chronic loss of autocrine VEGF induces the redundant expression of VEGF family members in CRC cells. Two CRC cell lines, HCT116 and RKO, were treated with an anti-VEGF mAb, which acts exclusively on secreted/extracellular VEGF, for 2 or 60 days (mAb-short or mAb-long cells, respectively), with non-immune control IgG for 2 or 60 days (IgG-short or IgG-long cells, respectively), or without any treatment (none). The expression levels of VEGF ligands (VEGF and PlGF) and VEGFRs (VEGFR-1 and -2) were measured by RT-qPCR. The mAb-short cell lines did not show a significant increase in the expression of any of the VEGF ligands or VEGFRs tested compared with the respective control IgG-short cells or untreated control cells (Figure 1A-D). In contrast, the mAb-long cells increased the expression of all of VEGF ligands and VEGFRs tested (approximately 2- to 2.5-fold) relative to the control IgG-long cells or untreated control cells (Figure 1A-D).

### Effect of chronic VEGF inhibition on apoptosis in CRC cells

**Figure 2:**
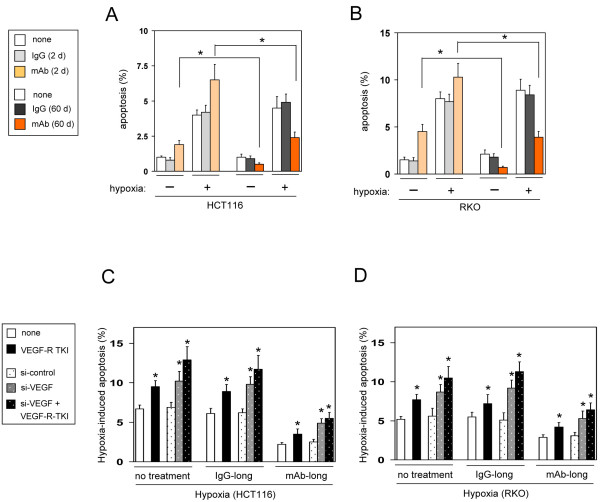
**Chronic blockade of secreted VEGF induces a resistance to apoptosis. **(**A**, **B**) Spontaneous and hypoxia-induced apoptosis. Cells that were treated without (none) or with an unimmunized IgG or an anti-VEGF mAb for 2 or 60 days were exposed to normoxia or hypoxia (~0.1% O_2_) for 3 days, then apoptotic cells were determined by TUNEL assay (n=5, means ± S.D.). (**C**, **D**) Effect of VEGFR-TKI or knockdown of *Vegf *mRNA on hypoxia-induced apoptosis in the mAb-long cells. Cells were untreated (none) or treated with VEGFR-TKI, or transfected with the indicated siRNA, or both for 3 days under hypoxic conditions. Then, apoptotic cells were determined by TUNEL assay. *P < 0.01., compared with the respective untreated control cells (none) (n=5, means ± S.D.).

As one of the major *in vivo* effects of VEGF inhibition is on angiogenesis and its contribution to tumor hypoxia, we examined sensitivity to hypoxia-induced apoptosis in the mAb-short and the mAb-long cell lines. Treatment with an anti-VEGF mAb for 2 days significantly increased spontaneous apoptosis under normoxia conditions, compared with control IgG-treated cells or untreated control cells (Figure 2A and B). These results demonstrate that autocrine/paracrine VEGF directly effected on and was a survival factor for these CRC cell lines as previously reported [7,11]. By contrast, the mAb-long cell lines showed significant resistance to spontaneous apoptosis (Figure 2A and B). These results suggest that the mAb-long cells, but not the mAb-short cells, had adapted to the loss of autocrine VEGF survival signal.

We then examined the effect of VEGF inhibition on hypoxia-induced apoptosis. After exposure to hypoxic conditions for 48 h, the mAb-short cells displayed a heightened degree of apoptosis compared with the respective control IgG-short cells or untreated control cells (Figure 2A and B). In contrast, the mAb-long cell lines showed a marked resistance to hypoxia-induced apoptosis (Figure 2A and B).

One possible mechanism for the adaptive resistance to apoptosis in the mAb-long cell lines is that redundant expression of VEGF family members compensated for the loss of the VEGF survival signal (Figure 1). To address this possibility, cells were treated with a VEGFR tyrosine kinase inhibitor (TKI), which inhibits both VEGFR-1 and -2. Treatment with VEGFR-TKI significantly increased hypoxia-induced apoptosis in the mAb-long cells, but did not completely abrogate their apoptotic resistance (Figure 2C and D). This result indicates that redundant pathways of VEGF family members did not completely compensate for the chronic blockade of the VEGF signal, and suggests that additional anti-apoptotic mechanism(s) may exist in the mAb-long cells.

We recently reported that the 5′UTR of *Vegf* mRNA induces resistance to apoptosis in HCT116 and RKO cells [15]. In addition, Lee et al. [16] reported that VEGF functions as an internal autocrine survival factor in human breast cancer cells through internally expressed VEGFR-1. These reports indicated the possibility that the 5′UTR of *Vegf* mRNA and/or intracrine VEGF may be essential survival factors in CRC cells. As shown in Figure 1A, expression of *Vegf* mRNA was increased more than 2-fold in the mAb-long cell lines relative to the respective control cells. Therefore, to inhibit both the 5′UTR of *Vegf* mRNA and intracrine VEGF, *Vegf* mRNA was knocked down with an siRNA targeting the exon 3 (si-*Vegf*), which is a common exon present in all splice variants. The knockdown efficiency of si-*Vegf* was 88% + 9% (n=4, means ± S.D.) compared with control siRNA. As expected, knockdown of *Vegf* mRNA increased hypoxia-induced apoptosis in the control IgG-long cells or untreated control cells (Figure 2C and D). By contrast, silencing of *Vegf* mRNA in the mAb-long cells slightly increased the rate of apoptosis, but the frequency of apoptosis still remained significantly lower than in controls (Figure 2C and D). These data suggest that the anti-apoptotic phenotype of the mAb-long cell lines is partially dependent on the *Vegf* mRNA 5′UTR and/or intracrine VEGF as well as on the compensatory pathways driven by VEGF family members. However, still other mechanisms must contribute to the apoptotic resistance.

**Figure 3:**
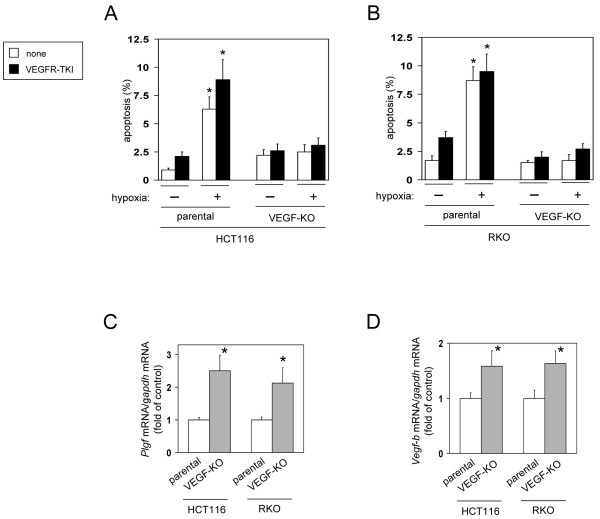
**Chronic and complete loss of all *Vegf *gene products augments resistance to hypoxia-induced apoptosis.** (**A**,**B**) Parental (VEGF^+/+^) or VEGF-KO (VEGF^-/-^) cell lines were exposed to normoxia for 2 days or hypoxia for 3 days in the absence (none) or presence of a VEGFR-TKI, then apoptotic cells were measured by TUNEL assay. *P < 0.01., compared with the respective normoxic control cells in each cell lines (n=6, means ± S.D.). (**C**,**D**) Expression levels of *Plgf *(**C**) and *Vegf-b *(**D**) were measured by quantitative RT-PCR (n=4, means ± S.D.). *P < 0.01., compared with parental (VEGF^+/+^) cells.

To further assess the effects of chronic and complete depletion of *Vegf* gene products (both *Vegf* mRNA and its protein) on hypoxia-induced apoptosis, we used two pairs of isogenic CRC cell lines (HCT116 and RKO) in which the *Vegf* gene was disrupted. HCT/VEGF-KO and RKO/VEGF-KO cells were generated by homologous recombination-mediated deletion of both *Vegf* alleles as described previously [17]. The loss of both *Vegf* mRNA and its protein expression was confirmed by RT-PCR (data not shown) and ELISA [17], respectively. Surprisingly, both VEGF-KO cell lines exhibited more resistance to hypoxia-induced apoptosis than parental cells (Figure 3A and B). This resistance was not reversed by treatment with a VEGFR-TKI (Figure 3A and B), although the VEGF-KO cell lines showed an increased expression of Plgf and VEGF-B (Figure 3C and D). These findings implicated additional adaptive survival pathways that are potently activated in VEGF-KO cell lines and are independent of the *Vegf* mRNA 5′UTR and intracrine or autocrine VEGF.

**Figure 4:**
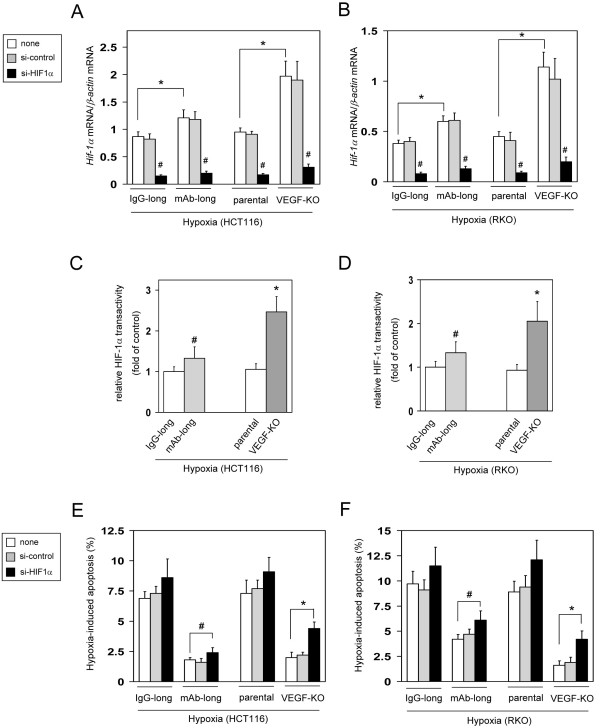
**HIF-1α is involved in apoptotic resistant phenotype in the mAb-long cells and VEGF-KO cells. **(**A**, **B**) Expression levels of *HIF-1α *mRNA in the mAb-long cells or VEGF-KO cells. Cells were untransfected (none) or transfected with control siRNA (si-control) or HIF-1α-targeting siRNA (si-HIF-1α) in HCT116 (**A**) or RKO cells (**B**), then they were exposed to hypoxia (~0.1% O_2_) for 36 h (n=6, means ± S.D.). *P < 0.01., compared with the respective untransfected cells (none) between IgG- and mAb-cells, or between parental and VEGF-KO cells. ^*#*^*P *< 0.01., compared with the respective untransfected cells (none) in each group. (**C**, **D**) Transcriptional activity of HIF-1α under hypoxic conditions in the mAb-long cells or VEGF-KO cells. Cells were transfected with a HIF-1α-dependent reporter (LucF) construct and a internal control reporter (LucR), then they were exposed to hypoxia (~0.1% O_2_) for 36 h in HCT116 (**C**) or RKO cells (**D**). The transcriptional activity of HIF-1α was determined by a dual luciferase assay (n=5, means ± S.D.). *P < 0.01., ^*#*^*P *< 0.05. (**E**, **F**) The levels of hypoxia-induced apoptosis in the mAb-long cells or VEGF-KO cells. Cells were untransfected (none) or transfected with si-control or si-HIF-1α in HCT116 (**E**) or RKO cells (**F**), then they were exposed to hypoxia (~0.1% O_2_) for 3 days. Apoptotic cells were measured by TUNEL assay (n=6, means ± S.D.). *P < 0.01., ^*#*^*P *< 0.05.

To further explore how the VEGF-KO cells became resistant to hypoxia-induced apoptosis in spite of their loss of all *Vegf* gene products, we focused on HIF-1α. HIF-1α is a critical regulator of many hypoxia responses, including resistance to apoptosis [18,19] and participates in resistance to VEGF inhibition, including *Vegf*-depleted tumor cells [20,21]. Expression levels and transcriptional activity of HIF-1α were up-regulated by approximately 2-fold in the VEGF-KO cells compared with the respective control cells under hypoxic conditions (Figure 4A-D). Knockdown of HIF-1α expression by RNAi (Figure 4A and B) caused an approximately 3-fold increase in hypoxia-induced apoptosis in the VEGF-KO cells, though the amount of apoptosis remained lower than that of the respective control cells (Figure 4E and F). Also in the mAb-long cells, expression levels and transactivity of HIF-1α were significantly up-regulated, and knockdown of HIF-1α modestly increased hypoxia-induced apoptosis compared with the respective IgG-long control cells (Figure 4A-F). These findings indicate that HIF-1α is involved in the anti-apoptotic phenotype of the VEGF-KO as well as mAb-long cell lines under hypoxia.

**Figure 5:**
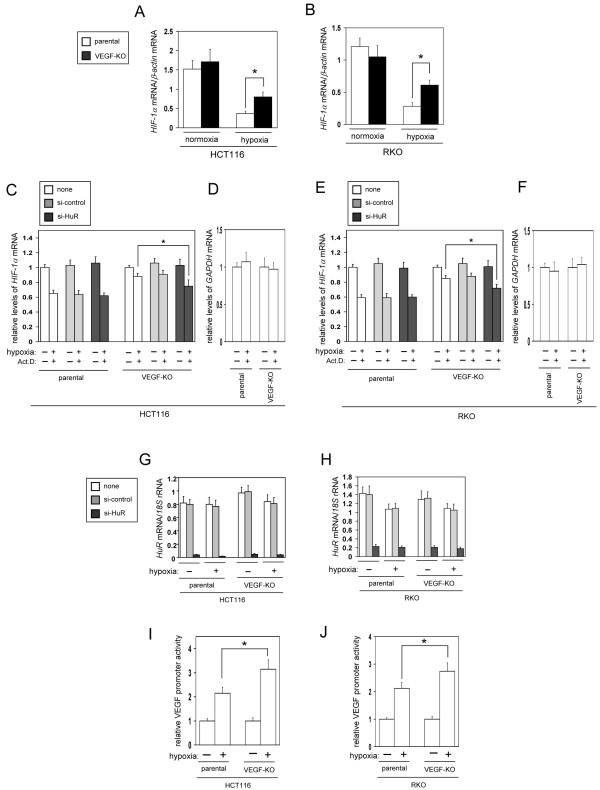
**Stability of *Hif-1α *mRNA is increased in VEGF-KO cells. **(**A, B**) Expression levels of *Hif-1α *mRNA in parental and VEGF-KO cell lines under normoxia and hypoxia. Cells were exposed to hypoxia (~0.1% O_2_) for 16 h. *Hif-1α *mRNA levels were determined by RT-qPCR (n=5, means ± S.D.). *P < 0.01. (**C-F**) *Hif-1α *mRNA stability was increased by HuR in VEGF-KO cells. Stability of *Hif-1α *(**C, E**) and *Gapdh *(**D, F**) mRNA was determined in cells transfected with the indicated siRNA in the presence of actinomycin D (Act. D) under hypoxia for 8 h, (n=5, means ± S.D.). *P < 0.01. (**G, H**) Knockdown efficiency of HuR mRNA. Cells were untransfected (none) or transfected with siRNA targeting HuR mRNA or control siRNA for 8 h, then they were exposed to hypoxia or normoxia for 16 h. HuR mRNA levels were determined by RT-qPCR (n=5, means ± S.D.). (**I, J**) VEGF promoter activity. Cells were transfected with a GFP reporter construct containing VEGF promoter sequence for 12 h, then they were exposed to hypoxia for additional 16 h. VEGF promoter activity was assessed by quantification of GFP reporter mRNA levels by RT-qPCR and normalized to the levels of Renilla luciferase mRNA (n=5, means ± S.D.). *P < 0.01.

We then examined how *Hif-1α* mRNA levels were increased in the VEGF-KO cells compared with the respective parental cells under hypoxia. There is evidence that the levels of *Hif-1α* mRNA are mainly regulated by mRNA stability mediated by HuR that binds the 3′UTR of *HIF-1α* mRNA and stabilize it [22]. Thus, we tested a stability of *Hif-1α* mRNA in VEGF-KO and their parental cells. The levels of *Hif-1α* mRNA under normoxic conditions were similar between VEGF-KO and their parental cells (Figure 5A and B). However, hypoxic treatment remarkably decreased *Hif-1α* mRNA levels in the parental cells compared with VEGF-KO cells (Figure 5A and B). In the presence of actinomycin D (Act. D) under hypoxic conditions, *Hif-1α* mRNA levels in the parental cells were more rapidly decreased than those in VEGF-KO cells (Figure 5C and E, open bars). For comparison, as seen Figure 5D and F, hypoxic treatment had no effect on *Gapdh* mRNA stability, which was used as a control transcript. These results indicate that *Hif-1α* mRNA is more stable in VEGF-KO cells than parental cells.

We further examined whether HuR is involved in the stability of *Hif-1α* mRNA in VEGF-KO cells. When HuR was knocked down using siRNA (knockdown efficiency was approximately 5-13% of control cells transfected with control siRNA; Figure 5G and H), *Hif-1α* mRNA levels in VEGF-KO cells were significantly decreased under hypoxic, but not normoxic, conditions, compared with the control siRNA-transfected cells (Figure 5C and E, closed bars). In contrast, *Hif-1α* mRNA levels in parental cells were not affected by knockdown of HuR (Figure 5C and E, closed bars). These findings indicate that HuR specifically participated in hypoxia-associated *Hif-1α* mRNA stabilization in VEGF-KO cells, but not in the parental cells.

The up-regulation of HIF-1α levels specifically observed in VEGF-KO cells suggests that VEGF-KO cells strive to activate *Vegf* mRNA transcription by increasing HIF-1α to adapt to chronic loss of VEGF. In fact, the promoter activity of *Vegf* mRNA was higher in VEGF-KO cells than in parental cells (Figure 5I and J). However, VEGF protein was not produced in the VEGF-KO cells (data not shown). Thus, the VEGF-KO cells also activated HIF-1α-dependent, but VEGF-independent, survival pathway(s) (Figure 4E and F).

### Chronic loss of VEGF increases spheroid formation by CRC cells

**Figure 6:**
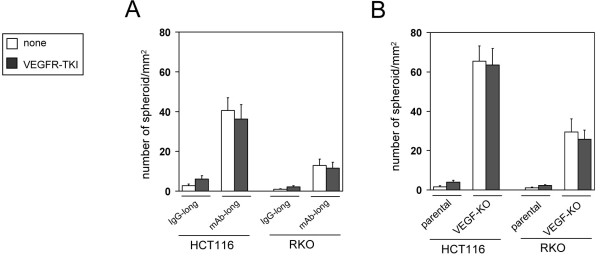
**Increase in spheroid formation ability in the mAb-long cells and VEGF-KO cells.** The mAb-long cells (**A**) or the VEGF-KO cells (**B**) were cultured for 6 days in a 3-D spheroid cell culture system in the absence or presence of VEGFR-TKI. Then, the number of spheroids were counted (n=5, means ± S.D.).

Multicellular spheroid culture provides an optimal model of hypoxia *in vitro*[23,24]. As the mAb-long and the VEGF-KO cell lines were resistant to hypoxia, we hypothesized that both cell lines would exhibit a higher ability to form multicellular spheroid. To test this hypothesis, each cell lines were cultured in a 3-D spheroid cell culture system. Control IgG-long cells and parental cells formed few spheroids (Figure 6A and B). Conversely, in accord with the observed apoptotic resistance, the mAb-long and the VEGF-KO cell lines showed a dramatically increased ability of spheroid formation, respectively, compared with the respective control cells (Figure 6A and B). Their ability was not suppressed by treatment with a VEGFR-TKI (Figure 6A and B). In proportion to the apoptotic resistant capacity, the frequency of spheroid formation was higher in the VEGF-KO cells than in the mAb-long cells (Figure 6A and B).

## Discussion

This study focused on the direct effects of VEGF inhibition on tumor cells using models of not only chronic blockade of secreted/extracellular VEGF derived from tumor cells (mAb-long cells), but also chronic depletion of all *Vegf* gene products (VEGF-KO cells). This design stands in contrast to other studies that have focused entirely on only extracellular VEGF. We found that chronic inhibition of extracellular VEGF by an anti-VEGF mAb resulted in resistance to hypoxia-induced apoptosis and an increased sphere formation ability in CRC cell lines. Surprisingly, the phenotypes observed upon inhibition of extracellular VEGF were further accelerated upon complete depletion of all *Vegf* gene products.

In response to chronic blockade of extracellular VEGF, redundant expression of PlGF was observed in the mAb-long cells. Many studies have similarly shown that inhibition of VEGF signaling *in vitro* or *in vivo* leads to compensatory increases in the expression of VEGF family ligands [2-5,11]. Treatment with VEGFR-TKI only partially suppressed the phenotypes observed herein, although a previous report by Samuel et al. showed that VEGFR-TKI almost completely abrogated the increased invasiveness of HCT116 cells chronically exposed to bevacizumab for 3 months [11]. This difference might result from the distinct experimental conditions between these studies. However, this discrepancy indicates that the phenotypic changes induced by chronic inhibition of extracellular VEGF did not necessarily depend on compensatory pathways activated by VEGF family ligands, and can most likely be attributed to other pathway(s).

Indeed, we elucidated that intracellular *Vegf* gene product(s) contributed to the apoptotic resistance in this model. The anti-apoptotic phenotype of the mAb-long cells was partially blocked by knockdown of *Vegf* mRNA. This finding indicated that tumor cells adapted to the chronic loss of the VEGF survival signal by means of intracellular functions of the 5′UTR of *Vegf* mRNA and/or intracrine VEGF protein, as we and others previously reported [15,16]. Thus, the mAb-long cells activated both compensatory pathways and intracellular pathway(s) involving *Vegf* gene products in response to chronic loss of extracellular VEGF.

Chronic loss of both extracellular and intracellular *Vegf* gene products (VEGF-KO cells) augmented the malignant phenotypes compared with the loss of only extracellular VEGF. These phenotypes were not suppressed by VEGFR-TKI, although a compensatory increase in VEGF family ligands were observed. This result is consistent with a previous report showing that the survival of VEGF-KO HCT116 cells was not affected by VEGFR-TKI [25]. These observations suggest that, relative to inhibition of only extracellular VEGF, chronic and complete depletion of all *Vegf* gene products may activate an additional adaptive mechanism(s) that is independent of compensatory pathways as well as the intracellular VEGF pathway(s). Thus, our findings demonstrate a complex intracellular role for VEGF signaling in cancer cells that may influence the clinical outcome of anti-VEGF therapy.

One of possible adaptive mechanisms may involve a HIF-1α-dependent pathway. The expression and activity of HIF-1α were increased in VEGF-KO cell lines, and knockdown of HIF-1α significantly suppressed the phenotypes of VEGF-KO cells. Many studies have established critical roles for HIF-1α in tumor cell survival and malignancy: i) HIF-1α is involved in repression of hypoxia-induced apoptosis in HCT116 and RKO cells *in vitro*[26]; ii) HIF-1α is required for VEGF-deficient tumor cell survival under hypoxic conditions *in vivo*[20], and iii) HIF-1α supports spheroid formation [18]. Most recently, two reports demonstrated that HIF-1α plays important roles in resistance to VEGF inhibition [20,21].

VEGF inhibition may produce two independent effects on tumor cells. The first is an “antiangiogenesis-dependent effect” that induces hypoxia through suppression of tumor angiogenesis. The increased hypoxia up-regulates HIF-1α expression and induces hypoxic selection of cancer cells and thus promotes their aggressiveness [14,18]. The second is an “antiangiogenesis-independent effect”, i.e., a direct effect on tumor cells. Inhibition of VEGF signaling in tumor cells directly induced malignant phenotypes through, at least in part, HIF-1α up-regulation [20,21]. These two effects may synergistically accelerate tumor malignancy *in vivo*, eventually resulting in resistance to anti-VEGF therapies.

Based on the present data and recent reports, it is possible that anti-VEGF therapies directly inhibit VEGF signaling in tumor cells, which may remodel tumor cell survival signal(s). In fact, recent reports clearly showed that VEGF signaling in tumor cells suppresses migration and invasion of tumor cells *in vivo*[27]; inhibition of VEGF signaling conversely accelerated migration and invasion *in vivo*[12,13]. These findings suggest that over the long term inhibition of VEGF, such remodeling result in adaptation to VEGF inhibition, and this adaptive response may represent one of potential mechanism of acquired resistance to anti-VEGF therapies.

VEGF initially held great promise as a therapeutic target, in fact, VEGF-targeting therapy has been shown to be very effective in certain tumor types, such as renal cell carcinoma [28,29]. However, the overall benefit of blocking VEGF activity in other solid tumors is marginal and has led to some skepticism in the field. Recently, Samuel et al. suggested that strategies to block VEGF signaling based on agents that neutralize secreted VEGF or inhibit its receptors may not block intracellular VEGF activities in tumor cells [25]. Based on the idea, the authors proposed that other methods that decrease or ablate intracellular VEGF, such as siRNA therapeutics targeting VEGF that can block all *Vegf* gene products, may provide new opportunities to improve current VEGF-targeting therapies. However, as demonstrated in the present study, depletion of all *Vegf* gene products actually enhanced tumor cell aggressiveness. Therefore, the use of drugs targeting VEGF/VEGFR as well as siRNAs targeting *Vegf* mRNA has the potential to promote tumor malignancy via an antiangiogenic-independent pathway. Therefore, molecular mechanism(s) activated by chronic loss of *Vegf* gene products will need to be elucidated to improve and further develop VEGF-targeting therapies.

## Conclusions

In this study, we elucidated that chronic inhibition of cancer cell-derived VEGF directly affected on tumor cells and accelerated their aggressiveness. Thus, these results suggest that VEGF-targeting drugs may directly induce resistance to anti-VEGF therapy.

## Competing interest

The authors declare that they have no competing interests.

## Authors’ contributions

NY carried out the cellular and molecular genetic studies and drafted the manuscript. KM, KN, and YK performed cellular studies. DLH and DTD established the VEGF-knockout HCT116 and RKO cell lines. TN and KR contributed to experimental design and helped to draft the manuscript. STK designed and directed the study, and helped to draft the manuscript. All authors read and approved the final manuscript.

## Pre-publication history

The pre-publication history for this paper can be accessed here:

http://www.biomedcentral.com/1471-2407/13/229/prepub
